# Wiggle and Shake: Managing and Exploiting Conformational Dynamics during Proteasome Biogenesis

**DOI:** 10.3390/biom13081223

**Published:** 2023-08-06

**Authors:** Daniel Betancourt, Tomiwa Lawal, Robert J. Tomko

**Affiliations:** Department of Biomedical Sciences, Florida State University College of Medicine, Tallahassee, FL 32306, USA; daniel.betancourt@med.fsu.edu (D.B.); tomiwa.lawal@med.fsu.edu (T.L.)

**Keywords:** 26S proteasome, ubiquitin, proteolysis, ATPase, macromolecular assembly, chaperone, conformation, dynamics

## Abstract

The 26S proteasome is the largest and most complicated protease known, and changes to proteasome assembly or function contribute to numerous human diseases. Assembly of the 26S proteasome from its ~66 individual polypeptide subunits is a highly orchestrated process requiring the concerted actions of both intrinsic elements of proteasome subunits, as well as assistance by extrinsic, dedicated proteasome assembly chaperones. With the advent of near-atomic resolution cryo-electron microscopy, it has become evident that the proteasome is a highly dynamic machine, undergoing numerous conformational changes in response to ligand binding and during the proteolytic cycle. In contrast, an appreciation of the role of conformational dynamics during the biogenesis of the proteasome has only recently begun to emerge. Herein, we review our current knowledge of proteasome assembly, with a particular focus on how conformational dynamics guide particular proteasome biogenesis events. Furthermore, we highlight key emerging questions in this rapidly expanding area.

## 1. The 26S Proteasome: A Central Protease for Regulated Intracellular Protein Degradation

In eukaryotic cells, biological processes as diverse as signaling, metabolism, and immune defense rely on the timely synthesis and removal of specific proteins from the cell while sparing most others. Similarly, the regulated removal of damaged or defective proteins maintains the overall quality of the proteome and protects cell and organismal health. In eukaryotes, most of these regulatory and quality control protein substrates are targeted for destruction by the ubiquitin–proteasome system (UPS) [1,2]. Proteins destined for degradation by the UPS are typically first modified via the covalent attachment of a small, highly conserved protein called ubiquitin (Ub) to one or more lysine residues in the protein. Attachment of additional Ub molecules to the initial Ub in turn permits the formation of a polyubiquitin (pUb) chain. This pUb chain can then serve as a signal for the recruitment of the modified protein to the 26S proteasome, an ATP-dependent protease complex responsible for destroying the substrate and releasing Ub for reuse. In this way, the 26S proteasome serves as the endpoint of the UPS.

**Figure 1:**
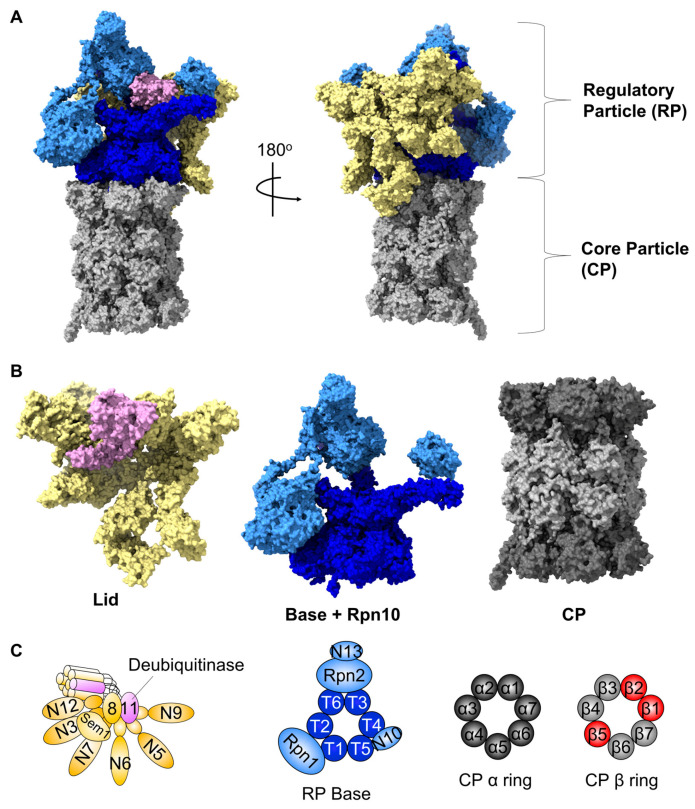
Structure of the canonical 26S proteasome and its three major subcomplexes. (**A**) the cryo-electron microscopy structure of the 26S proteasome in the resting/ground state is shown (PDB: 6FVT). The lid subcomplex is shown in yellow, the base ATPase ring is shown in dark blue, and the non-ATPase subunits, including Rpn10, are shown in light blue. The core particle (CP) is shown in grey. (**B**) the three major subcomplexes of the proteasome are shown individually. Coloring is as in (**A**) for the lid and base, and the α and β rings of the CP are shown in dark and light grey, respectively. (**C**) Cartoon depictions of the subunit compositions of each subcomplex are shown. The major deubiquitinase of the proteasome, Rpn11, is shown in plum, and the three catalytic β subunits are shown in red.

At approximately 2.5 megadaltons, the 26S proteasome is an unusually large and complex protease. Although several variants of the proteasome harboring slight differences in composition and function exist, this review will focus on the canonical form of the 26S proteasome (hereafter proteasome). The proteasome consists of a 19S regulatory particle (RP) that docks onto one or both ends of the barrel-shaped 20S core particle (CP) (Figure 1A). The RP is responsible for recognition of incoming substrates, removal of the pUb targeting signal, unfolding them into linear polypeptide strings, and translocating them into the central chamber of the CP [1]. The CP in turn cleaves the unfolded substrate into short peptides that can then be further proteolyzed to yield free amino acids for new protein synthesis.

The RP can be further divided into two subcomplexes: the lid and the base (Figure 1B) [3]. The lid consists of nine regulatory particle non-ATPase (Rpn) subunits; Rpn3, Rpn5–9, Rpn11, Rpn12, and Rpn15/Sem1 (Figure 1C). Rpn11 is a metalloprotease and the sole intrinsic deubiquitinating enzyme present in the proteasome [4,5]. The central unit within the base is a hexameric ring formed by six AAA+ family ATPase subunits, named Rpt1–6 and arranged Rpt1–Rpt2–Rpt6–Rpt3–Rpt4–Rpt5 (Figure 1B,C) [6]. This ATPase ring is adorned with three non-ATPase subunits: Rpn1, Rpn2, and Rpn13. One additional subunit, Rpn10, lies at the interface between the lid and base and helps to stabilize their interaction. Rpn1, Rpn10, and Rpn13 contain well-characterized Ub-binding motifs that capture polyubiquitinated substrates [7,8,9,10], whereas the six Rpt subunits use the energy of ATP binding and hydrolysis to exert a pulling force on substrate proteins, unfolding them and translocating them through a narrow central pore into the CP. The CP consists of four heteroheptameric rings axially stacked in an α–β–β–α ring conformation (Figure 1B). The α rings, composed of seven α subunits (α1–α7), form the outer rings in the stack, whereas the β rings, composed of seven β subunits (β1–β7), make the inner rings in the stack of the 20S proteasome (Figure 1C). The β1, β2, and β5 subunits contain caspase-like, tryptic-like, and chymotryptic-like activities, respectively. These subunits catalyze the hydrolysis of unfolded proteins into short peptides.

Whereas the catalytic activities of the proteasome have been carefully studied since its initial discovery in the mid-1980s [11,12], the assembly of this unusually large cellular machine is in its comparative infancy. Already, investigation of proteasome biogenesis has revealed numerous important paradigms for macromolecular assembly in general and will likely continue to do so for many additional years. Here, we review the current knowledge on proteasome biogenesis, with a particular focus on recent advancements in how conformational dynamics control key assembly steps.

## 2. An Overview of Proteasome Biogenesis

The assembly of the proteasome has been pieced together over the past 30 years through a combination of molecular, structural, and biochemical studies performed across prokaryotes, archaea, and eukaryotes [13]. In eukaryotes, whose proteasomes will be the major focus of this review, most of the work has been carried out in budding yeast and cultured human cells. Given the ~66 individual polypeptide chains present in a mature proteasome, an astronomical number of subunit assembly sequences is theoretically possible. However, most evidence currently supports a highly restricted number of possible subunit assembly sequences. Indeed, protein complexes are generally under evolutionary selection to assemble via ordered pathways rather than via a random stochastic process [14]. Among other benefits, such ordered assembly likely reduces the risk of misassembly events and accelerates the production of mature complexes from its constituent subunits. Ordered assembly likely also reduces the risk of assembly deadlock, an event in which the simultaneous use of multiple assembly pathways can lead to the depletion of free subunits necessary for completion of assembly via any individual pathway.

Order during proteasome biogenesis is enforced, at least in part, by a combination of intrinsic subunit affinities, avid interactions among subunits within assembly intermediates, chaperone-like functions of unstructured regions of subunits, and the concerted efforts of several evolutionarily conserved assembly factors [2,13]. Generally, these assembly factors enhance the natural propensity of proteasome subunits to self-assemble, but in some cases may be essential for proper formation of particular intermediates [15,16,17,18,19,20,21,22,23,24]. In agreement with this, deletion of individual proteasomal assembly chaperones is typically tolerated with only some modest impact on assembly, whereas co-deletion of multiple assembly factors or combination with hypomorphic mutations in proteasome subunits often lead to growth defects ranging from modest to synthetic lethality [15,16,17,18,19,20,21,22,23,24]. As described below, these unstructured regions and assembly factors often serve to reinforce intersubunit interfaces that are comparatively weak in the context of assembly intermediates, or to instead restrict the inappropriate interaction between particular subunits or assembly intermediates. Although some studies suggest that that the three major subcomplexes of the proteasome—the CP, the lid, and the base—undergo interconnected assembly [21,25,26,27], most evidence to date indicates these three subcomplexes preferentially assemble independently of one another, followed by their stepwise association to form mature proteasomes. Below, we provide a summary of what is known regarding the assembly of each subcomplex.

### 2.1. CP Assembly

In simpler organisms such as actinobacteria and archaea, the CP is typically composed of homomeric α and β rings containing seven copies of a single α or β subunit, respectively. Formation of the CP typically occurs via one of two pathways. In actinobacteria, α and β subunits first associate with one another to yield heterodimers, with seven heterodimers then associating laterally to yield a species composed of stacked α and β rings known as half-proteasomes [28,29,30]. Two half-proteasomes then associate to yield a species called a pre-holoproteasome (PHP). Formation of the PHP promotes the autocatalytic cleavage of propeptides present at the N-termini of the catalytic β subunits, yielding mature CP. This autocatalytic cleavage step serves to restrict proteolytic activity during the assembly process and thus prevent premature, unregulated degradation of proteins. In Archaea, a second pathway occurs that is more similar to that observed in eukaryotes. In this path, α rings first form from seven α subunits, and this α ring then serves as a scaffold for the formation of the β ring, yielding a half-proteasome [31,32]. The dimerization of half-proteasomes and maturation of the active sites then proceed as for the first pathway.

**Figure 2:**
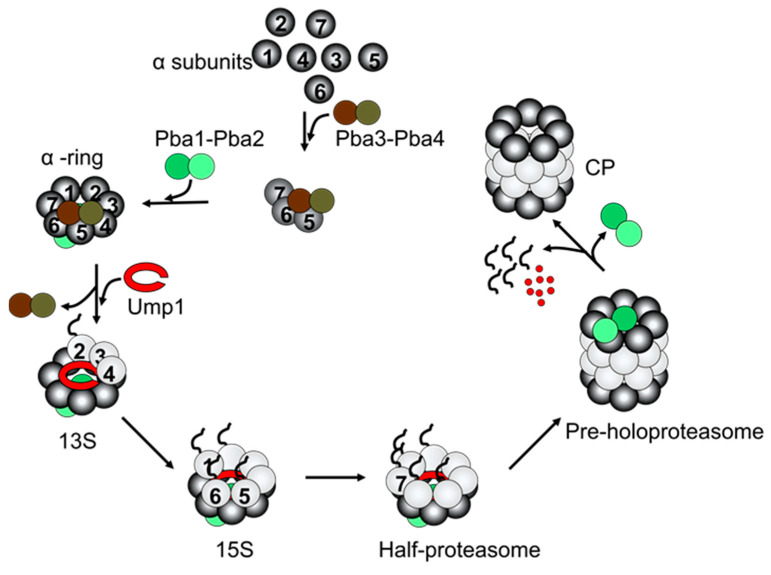
Core particle assembly pathway. β subunit propeptides are shown as squiggly lines. Early steps in α ring assembly are not entirely clear, but likely involve formation of an α5–α6–α7 complex that may also contain α1 and/or α4, bound by Pba3–4 [21,23,24,37,38,39,40,41]. Pba1–2 associate prior to or concurrently with completion of α ring assembly, which in turn forms a scaffold upon which the β subunits assemble. Ump1 stabilizes incoming β subunits via direct contacts, and also serves to retain Pba1–2 via interaction with the Pba1 N-terminus, which snakes through the central pore of the α ring. Docking of β2, β3, and β4 onto the assembled α ring creates the 13S intermediate. Docking of β5, β6, and β1 onto the 13S intermediate yields the 15S intermediate. Incorporation of β7 is the rate-limiting step for CP assembly and yields a complete half-proteasome. Dimerization of half-proteasomes forms the pre-holoproteasome, which undergoes autocatalytic propeptide processing and destruction of Ump1 to yield the mature CP. Pba1–2 is thought to be released from the mature proteasome via a combination of α ring constriction and loss of interactions with the propeptides and Ump1.

In eukaryotes, CP assembly is more complex, owing to the diversification of subunits present in the α and β rings. All eukaryotic proteasomal CPs studied to date contain at least seven distinct α and seven distinct β subunits, each encoded by a unique gene. Furthermore, each individual α or β subunit must occupy a specific location within its respective ring to yield a mature and functional proteasome. It is generally agreed that in eukaryotes, CP assembly occurs via the formation of an α ring containing the α1–α7 subunits, which is then loaded with β subunits β2, βb3, and β4 (Figure 2). This species is commonly referred to as the 13S intermediate [33], based on its sedimentation coefficient in ultracentrifugation experiments. Docking of β1, β5, and β6 occurs next, yielding the 15S intermediate, followed by incorporation of β7 to complete β ring assembly, yielding a half-proteasome [34,35]. Incorporation of β7 is generally considered to be rate-limiting for CP assembly, and is facilitated by its long C-terminal tail [35]. Two half-proteasomes then dimerize to yield a PHP that undergoes autocatalytic propeptide cleavage to produce a mature proteasome [36].

Although α ring formation initiates CP assembly, the earliest events are perhaps the least understood. At present, no α ring intermediates smaller than a fully formed ring have been directly and confidently observed in vivo. Certain combinations of α subunits bind one another in vitro when coexpressed in *E. coli* [21,38,39,42,43], suggesting that some self-assembly of subunits may occur. In many cases, these recombinant assembly products bear subunit compositions or arrangements that are unlikely to be physiological [24,38,39], strongly implying that α ring formation relies, at least in part, on extrinsic regulatory elements.

Indeed, α ring formation is facilitated by two sets of proteasome-specific chaperones, Pba1–2 and Pba3–4 (PAC 1–2 and PAC3–4 in metazoans) (Figure 2) [21,22,24,35,40]. Pba1–2 is a heterodimeric complex that guides assembly of the heptameric ring via dedicated interactions with each of the seven α subunits, and is retained through subsequent stages to prevent formation of off-pathway assembly products and α ring dimers [24]. By virtue of its binding to the RP-facing surface of the α ring, it also prevents premature RP–CP binding via steric interference. Recent cryo-electron microscopy (cryo-EM) structures of the 13S and pre-15S intermediates have revealed that the Pba1–2 heterodimer is positioned such that Pba1 lies over the α5–α6 interface with Pba2 positioned over the α ring’s central pore [44,45]. The N-terminus of Pba1 snakes beneath Pba2 and extends into the central α ring pore, thereby occluding premature access of substrates to the proteolytic chamber in later assembly stages.

The assembly role(s) of the Pba3-4 heterodimer is less understood, but it appears to function only during the earlier stages of CP assembly, as it is absent from the 13S intermediate (Figure 2) [44]. Genetic suppression of Pba3 or Pba4 in yeast or human cells results in the formation of non-canonical proteasomes containing a second copy of α4 in place of α3, suggesting a role for this heterodimer in α ring formation [21,46]. For further discussion of α4–α4 proteasomes the reader is directed to a recent review [2], although it is worth noting here that these non-canonical proteasomes have been reported in multiple organisms, may be more abundant in certain cancers [47], and can convey enhanced resistance to certain stresses [21].

The mechanism by which Pba3–4 ensures incorporation of a single copy of α4 is unknown, but may involve suppression of inappropriate subunit interactions. Pba3–4 associates most strongly with free α5 subunit in vitro [21,40], and has been observed associated with intermediates up to the 13S stage. Like Pba1–2, Pba3–4 binds primarily to the assembling α ring, but on the opposite surface as Pba1–2 [40]. No significant contacts are anticipated with α3 based on homology modeling [40], suggesting that Pba3–4 does not directly recruit α3 into the assembling α ring. Instead, most evidence to date suggests a mechanism in which it functions by suppressing inappropriate interaction between α2 and α5, thereby ensuring α4–α5 association [37]. Deletion of *PBA4* in yeast results in the formation of an aberrant assembly product resembling the 13S intermediate [37], but lacking α4 and containing twice the normal α2 content of a canonical α ring. Similarly, α2 interacts directly with α5 in vitro, and overproduction of α1 in yeast suppressed the formation of non-canonical proteasomes in *pba3Δ* cells [47], presumably by acting as a sink for α2 to limit aberrant α2–α5 association. Later in CP assembly, Pba3–4 has been proposed to ensure ordered incorporation of β subunits until it is sterically displaced by the incoming β4 subunit [34].

Once formed, the α ring functions as a template upon which β subunits can coalesce. Ump1, the first-discovered proteasomal assembly chaperone, plays a role in the structural integrity of early β subunit-containing assembly intermediates [44,45,48]. Recent cryo-EM structures suggest this is by reinforcing the α ring–β subunit interface during β ring assembly and by providing additional stabilizing contacts with incoming β subunits. Ump1 is first detected as part of the 13S intermediate, and remains bound until the completion of CP assembly, becoming encapsulated inside the maturing CP through the final activating step [48].

The subsequent addition of β1, β5, and β6 gives rise to the 15S intermediate [34]. The final subunit to be incorporated in yeast and mammals is the β7 subunit, completing the formation of a half-proteasome [35]. The half-proteasome then dimerizes to yield the PHP. The final step in proteasome biogenesis is the autocatalytic processing of propeptides on β subunits β1, β2, β5, β6, and β7 [36]. Propeptide processing generates the catalytic N-terminal threonine nucleophiles of β1, β2, and β5 necessary for substrate proteolysis [36], and promotes the destruction of the encapsulated Ump1 chaperone by the newly active CP [48]. This final maturation step also triggers the release of Pba1–2 from the α ring surface, rendering the CP competent for the binding of regulators such as the RP.

### 2.2. RP Assembly

Like CP assembly, the assembly of the RP has been pieced together largely based on the subunit composition of metastable complexes observed to accumulate in yeast cells harboring deletions or hypomorphic mutations in RP subunits or chaperones. These metastable complexes have generally been presumed to be assembly intermediates, and some studies have demonstrated that they can be incorporated into larger assemblies [20,49,50,51]. Most of the literature indicates that the two main subcomplexes of the RP—the lid and base—are able to assemble independently both of the CP and of one another, although some reports suggest that mixed intermediates containing both lid and base subunits can occur and assemble productively into the RP, suggesting that more than one assembly pathway may exist [26].

**Figure 3:**
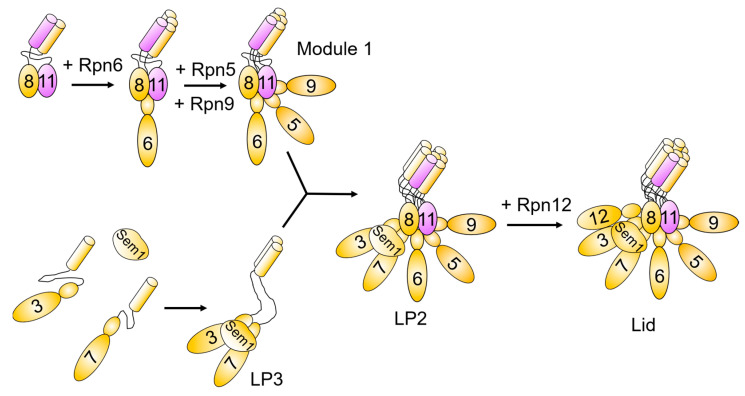
Lid subcomplex assembly pathway. Lid assembly in yeast proceeds via formation of Module 1 and lid particle (LP) 3, which are composed of Rpn5/6/8/9/11 and Rpn3/7/Sem1, respectively. Module 1 and LP3 then associate to form LP2. Incorporation of Rpn12 into LP2 yields the fully assembled lid subcomplex. Some limited evidence suggests lid formation may proceed via a distinct subunit incorporation sequence in humans [56].

Our knowledge of lid assembly comes almost entirely from studies in budding yeast [49,50,51,52,53,54,55,56], and in contrast to the CP and the base, the lid appears to assemble without the need for any dedicated assembly factors. In yeast, lid assembly begins with the formation of two complementary precursors via parallel paths (Figure 3) [50,52,53,54]. These precursors then combine, followed by joining of one final subunit to form the mature subcomplex. One path is initiated by dimerization of Rpn8 and Rpn11, which then recruits Rpn6 [52]. This trimeric complex then recruits Rpn5 and Rpn9 to form an intermediate referred to as Module 1 [54]. In the second path, Rpn3 and Rpn7 are tethered together by Sem1 to form an intermediate called lid particle 3 (LP3) [50]. Module 1 and LP3 then associate forming lid particle 2 (LP2). Finally, association of Rpn12 with LP2 completes the mature lid subcomplex [49,51].

The order of assembly events within the lid is driven primarily by a rigid helical bundle formed from α-helices present at the C-termini of all lid subunits except for Rpn15/Sem1 [52]. This dependence on the helical bundle is presumably due to the relatively limited surface area buried between the bodies of adjacent lid subunits, which is necessary for the flexing and repositioning of the lid that occurs during substrate catalysis [57,58]. As lid subunits assemble, their C-termini associate and create avid interaction sites for the C-termini of other lid subunits, thereby serving to regulate the sequence of subunit addition. This has been best studied for the final lid subunit to incorporate, Rpn12. The C-terminal helix of Rpn12 docks into a crevice formed by the C-terminal helices of virtually all other lid subunits [49,52]. The helices directly interacting with the Rpn12 helix are contributed by subunits of both LP3 and Module 1, rendering its stable incorporation dependent on the hierarchical assembly of these intermediates. Rpn12 thus serves as a sensor for the assembly state of the lid, docking stably only after successful incorporation of all other subunits has taken place.

Sem1, the sole lid subunit not contributing a helix to the helical bundle, serves a chaperone-like role during an early stage of lid assembly [50]. Sem1 is an intrinsically disordered protein, and the smallest subunit of the proteasome at ~10 kDa. Although Sem1 is not required for the structural integrity of the mature proteasome, it serves a key role by physically tethering lid subunits Rpn3 and Rpn7 together until their relatively weak interface can be reinforced by interactions with other lid subunits from Module 1. Sem1 utilizes two conserved charged regions separated by a flexible, poorly conserved linker region, to interact with Rpn3 and Rpn7 during this assembly function. Interestingly, Sem1 moonlights in several other multisubunit complexes, but based on available molecular structures, its deployment of these charged regions to stabilize a subunit–subunit interaction as it does in the proteasome appears to be unique.

Formation of the base requires the arrangement of the six Rpt ATPase subunits into a heterohexameric ring in the order Rpt1–Rpt2–Rpt6–Rpt3–Rpt4–Rpt5 [6], and adornment of the ring at specific positions by the non-ATPase subunits Rpn1, Rpn2, and Rpn13. Heterologous expression experiments have indicated that the six ATPase subunits do not encode all of the information necessary to assemble properly on their own [59], and work from numerous groups in yeast and mammalian cells have identified a collection of dedicated assembly chaperones, discussed below, that help coordinate formation of the base [15,16,17,18,19,20]. Biogenesis of the base appears to initiate with the pairing of the ATPases into Rpt1–Rpt2, Rpt3–Rpt6, and Rpt4–Rpt5. This process is mediated primarily by the N-terminal coiled coil domains of the ATPases and is facilitated by assembly chaperones. Each ATPase dimer is associated with at least one dedicated assembly chaperone. Four dedicated base assembly chaperones and one stress-induced chaperone have been identified to date [60], although the role of the stress-induced chaperone in proteasome biogenesis has recently been challenged [61].

**Figure 4:**
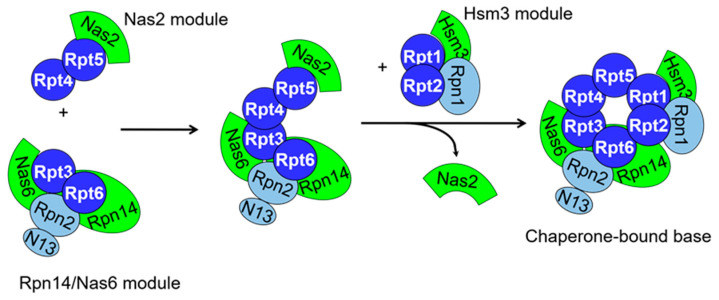
Yeast base assembly pathway. Base assembly initiates with the formation of Rpn14/Nas6, Nas2, and Hsm3 modules independently of one another. In yeast, the Rpn14/Nas6 and Nas2 modules first associate as shown. Nas2 dissociates from this intermediate immediately prior to or concurrently with incorporation of the Hsm3 module to yield a chaperone-bound base subcomplex that is competent for incorporation into mature proteasomes. In this cartoon, Rpn2 and Rpn13 are shown present in the initial Rpn14/Nas6 module, but the exact point(s) where these subunits enter the assembling base is uncertain. Also, the order of module association in humans appears to differ from that of yeast based on subunit knockdown experiments.

Generally, each dedicated chaperone binds to the C-terminal domain of a particular Rpt subunit to exert its functions. Specifically, Nas6 (p28/gankyrin in mammals) and Rpn14 (PAAF1) associate with Rpt3 and Rpt6, respectively; Hsm3 (S5b) associates with Rpt1; and Nas2 (p27) associates with Rpt5. The resultant Nas6–Rpt3–Rpt6–Rpn14, Hsm3–Rpt1–Rpt2, and Rpt4–Rpt5–Nas2 complexes are often referred to as the Rpn14/Nas6, Hsm3, and Nas2 modules, respectively (Figure 4). Once formed, these three modules associate stepwise to form the ATPase ring. Rpn1 can associate with the Hsm3 module prior to this stepwise association [15,16,17,18,19,20,62], but whether Rpn2 and Rpn13 similarly associate with the Rpn14–Nas6 module prior to interaction with other modules is not yet known. Rpn2 and Rpn13 can be readily co-precipitated with an assembly intermediate consisting of the Rpn14/Nas6 and Nas2 modules, however, suggesting they associate prior to completion of ATPase ring assembly [63].

Nas2 binds the three most terminal C-terminal residues of Rpt5 via its PDZ domain [64,65]. As these 3 C-terminal amino acids form a motif that docks into the surface of the CP in mature proteasomes [66,67], it has been suggested that a major function of Nas2 is to prevent premature association of Rpt4–Rpt5 with the CP [16,17,68]. The structure of Nas2 bound to the Rpt5 C-terminal helical domain indicates that Nas2 blocks the incoming Hsm3 module via steric conflict with Rpt1 [64]. This suggested that Nas2 must dissociate prior to Hsm3 module incorporation and likely enforces ordered association of the three modules (Figure 4). Indeed, immunoprecipitation of Nas2 from yeast cell extracts copurifies all subunits of the Nas2 and Rpn14/Nas6 modules (including their associated chaperones), but no subunits or chaperones of the Hsm3 module [6], strongly suggesting that these modules associate prior to incorporation of the Hsm3 module. In contrast, experiments in human cells have suggested pre-association of the Rpn14/Nas6 and Hsm3 modules, followed by incorporation of the Nas2 module to yield a chaperone-bound base precursor [19]. Thus, whereas the basic modules are identical from yeast to humans, it appears that their association sequence may vary from species to species.

Nas6 and Rpn14 associate with Rpt3 and Rpt6, respectively. Both are thought to antagonize base–CP premature interaction by binding to the C-terminal domains of their cognate ATPases, although the evidence of this for Rpn14 is currently rather limited [16,17]. Similarly, some limited evidence indicates these chaperones may help to stabilize the Rpn14/Nas6 module until it is incorporated into higher order assembly intermediates. In contrast, a role for Hsm3 as an assembly hub for Rpt1, Rpt2, and Rpn1 to yield the Hsm3 module is better supported [62]. In addition to its chaperone function, it likely prevents premature docking of the Hsm3 module and/or the base complex with CP via steric hindrance [68]. Association of the fully assembled lid and base then occurs, which is thought to trigger recruitment of the ubiquitin receptor Rpn10 to reinforce their interface and yield a chaperone-bound RP intermediate. Upon docking of this intermediate on the mature CP, Rpn14, Nas6, and Hsm3 are released, yielding a mature and catalytically active proteasome.

## 3. Conformational Dynamics Provide Challenges and Opportunities during Proteasome Assembly

Although there has recently been an explosion in our knowledge of the conformational dynamics of the proteasomal proteolytic cycle [57,58,69,70,71,72,73,74,75,76,77,78,79], our understanding of conformational dynamics during proteasome biogenesis is rather poorly developed. From a theoretical perspective, however, conformational dynamics provide both challenges to the assembly of multisubunit complexes, as well as several potential opportunities that can be exploited to ensure the timely, efficient, and faithful biogenesis of these macromolecular assemblies. In the following sections, we describe these challenges and opportunities, and highlight some examples of how they are implemented to regulate biogenesis of the proteasome.

Conformational dynamics can in principle be considered both in terms of their impacts on a protein’s three-dimensional structure, and also in terms of the timescales over which these structural movements occur. Highly localized movements within a given protein, such as amino acid side chain rotations or formation of secondary structural elements, typically occur over nanosecond timescales. In contrast, rearrangement of tertiary structural elements leading to largescale conformational changes within a protein are typically much slower, often occurring over millisecond or second timescales [80]. Extremely rapid events such as side chain rotamer exchange likely influence subunit interactions, but thus far have not been carefully investigated for the proteasome. Instead, most studies have focused on larger conformational changes occurring over millisecond to second timescales [81,82]. Thus, from here forward, our use of the terms “conformational changes” or “conformational dynamics” will refer to these larger scale tertiary structural rearrangements of subunits or complexes unless otherwise indicated.

### 3.1. Conformational Matching as A Requirement for Association of Assembly Intermediates

Conformationally dynamic subunits or assembly intermediates can pose challenges for macromolecular assembly if one or more conformational states assumed by a given intermediate is incompatible with a given assembly step. An elastic collision between two binding partners gives perhaps the simplest example of how conformational dynamics can influence the assembly of a macromolecular structure. If one were to imagine two dynamic conformational states that each partner can adopt–one in which they are assembly competent, and another in which they are assembly incompetent, then only collisions between partners in which both are in the assembly competent state would be anticipated to yield a productive assembly event. In contrast, events in which only one (or neither) partner has assumed the assembly competent state would be nonproductive. Put another way, all interaction partners must assume conformations that are capable of meshing with one another productively upon collision.

Perhaps the best example of such conformational matching as a means to control proteasome assembly is the role of Rpn12 in promoting a conformational state of the lid that is competent for docking with the base subcomplex. As described above, Rpn12 is the final lid subunit to incorporate, and it had been previously demonstrated that the LP2 intermediate, which only lacks Rpn12, cannot stably dock onto the base subcomplex unless Rpn12 is present to complete lid formation [49,51]. This was somewhat surprising as Rpn12 adopted a peripheral location within the lid and does not make direct contact with the base.

**Figure 5:**
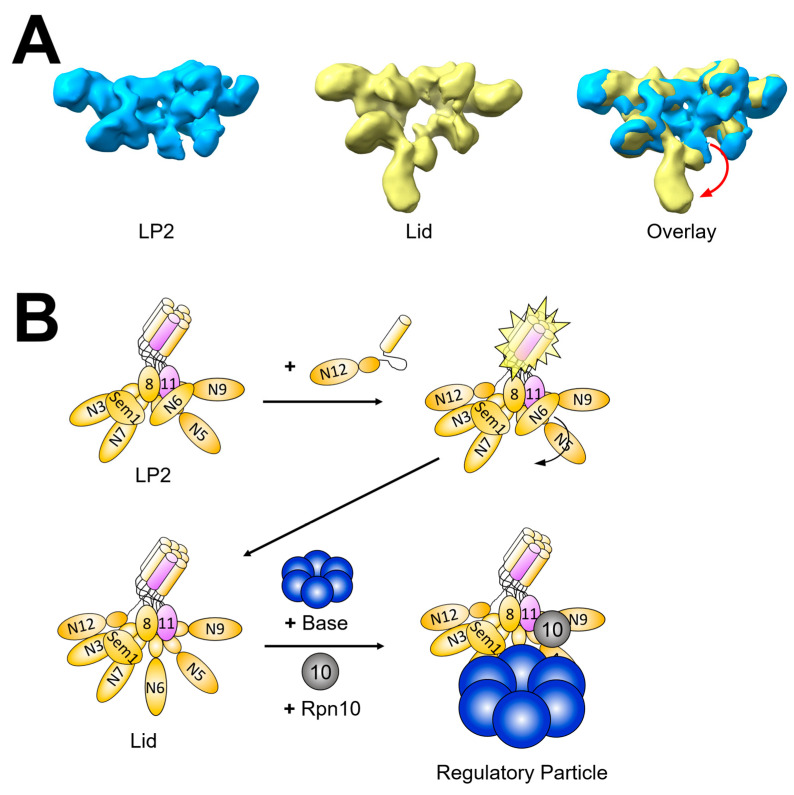
Conformational changes in the assembling lid license it for interaction with the base to yield the mature regulatory particle. (**A**) the negative stain electron microscopy densities for LP2 (EMD-3136; blue) and the free lid (EMD-1993; yellow) are shown, with their superimposition shown on the right. A red arrow indicates the change in density, presumed to be the N-terminal domain of Rpn6 based on crosslinking data [49], that occurs upon incorporation of Rpn12 into the assembling lid. (**B**) repositioning of Rpn6 reveals the central palm of the hand-like lid subcomplex and permits stable docking with the base to yield the regulatory particle.

A quantitative cross-linking mass spectrometry and electron microscopy study revealed that incorporation of Rpn12 induces significant conformational changes in the nascent lid, transitioning from a compact and autoinhibited ‘closed’ state observed in LP2 to an open and flexible state that can effectively interact with the base [49]. Comparison of the crosslinking profile for LP2 and lid revealed two main conformational differences. The first was in the positioning of the Rpn8–Rpn11 module relative to Sem1, Rpn3, and Rpn7, consistent with a more closely packed arrangement in LP2 compared to the lid. The second was in the positioning of the finger-like N-terminal domain of Rpn6, which was compacted toward the center of LP2, but extended in the context of the lid (Figure 5A, red arrow). This extension, coupled with repositioning of the Rpn8–Rpn11 heterodimer described below [83], exposes key surfaces that contact the base, permitting their stable interaction. Importantly, this rearrangement could be triggered by the addition of recombinant Rpn12 or the isolated C-terminal helix that contributes to the helical bundle, providing an explanation for regulation of lid–base assembly by Rpn12 (Figure 5B) [49].

A second example is provided by the base assembly chaperone Nas6. Nas6, by virtue of binding the C-terminal helical domain of Rpt3 [84], is located in close quarters to both the surface of the CP α ring and to the lid subunits Rpn5 and Rpn6. As for many AAA+ family ATPases [85], the positioning of the C-terminal helical domain of Rpt3 to which Nas6 binds undergoes an up-and-down movement in response to nucleotide binding and/or hydrolysis within the ATPase ring. This repositioning of the C-terminal helical domain brings Nas6 into steric conflict with the CP or with Rpn5/Rpn6 depending on the nucleotide state of the base [86,87]. In this manner, the nucleotide state may control the order in which the lid, base, and CP assemble to yield mature proteasomes. This is discussed in more detail below.

### 3.2. Conformational Restriction of Catalytic Activity during Proteasome Assembly

The mature, doubly RP-capped proteasome contains ~20 catalytic centers with deubiquitinating, unfoldase, and protease activities. In the context of the mature proteasome, these activities are carefully controlled to avoid spurious proteolysis via sequestration of the peptidase active sites in the center of the CP, via enzymatic coupling of the deubiquitinating activity to substrate unfolding and translocation [88], or via the requirement that a substrate be held in very close vicinity to the base ATPase pore via the prior capture of its polyubiquitin chain for engagement and unfolding. However, these safety mechanisms cannot always be implemented in the context of proteasomal assembly intermediates, necessitating mechanisms to restrict particular activities until assembly has been completed. For the CP proteolytic centers, peptidase activity is restricted via the production of the catalytic subunits as proproteins with N-terminal inhibitory propeptides. These propeptides are autocatalytically removed upon completion of CP assembly [36]. In contrast, suppression of the enzymatic activities of the lid and base instead relies on conformational mechanisms to restrict their respective functions.

One of the first examples of such conformational restriction of catalysis was shown for the deubiquitinating lid subunit Rpn11. When produced recombinantly as a heterodimer with its binding partner Rpn8, Rpn11 exhibits modest but highly promiscuous deubiquitinating activity [89]. However, its activity is reduced ~four-fold in the context of the isolated lid subcomplex [83]. Once the lid incorporates into the fully assembled 26S proteasome, Rpn11 activity is again enhanced [90]. These observations suggest that Rpn11 activity is restrained in the context of the lid subcomplex, and relieved upon incorporation into proteasome holoenzymes. Indeed, although the Rpn11/Rpn8 heterodimer has not been observed to accumulate appreciably in cells, substantial free lid has been observed [51], necessitating some regulatory mechanism to prevent spurious deubiquitination.

**Figure 6:**
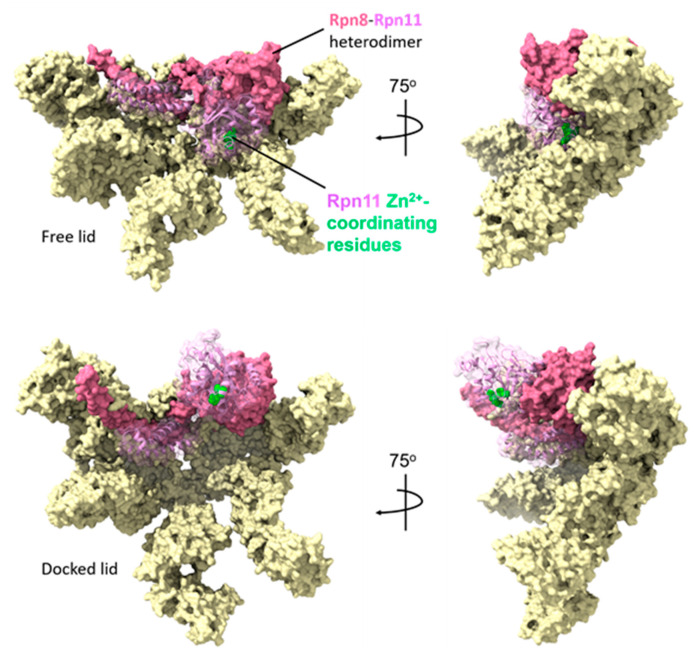
Autoinhibition of Rpn11 within the free lid is released upon incorporation into the mature proteasome. The cryo-electron microscopy structures of the free lid (PDB: 3JCK) and of the lid in the context of the mature proteasome (PDB: 6FVT) are shown. Subunits Rpn3, 5, 6, 7, 9, 12, and Sem1 are shown in pale yellow, whereas the Rpn8–Rpn11 heterodimer is shown in pink and plum, respectively. The ribbon structure of Rpn11 with the amino acids that coordinate the catalytic Zn^2+^ ion is shown in green space-filling representation. As shown, in the free lid, the Rpn8–Rpn11 heterodimer is folded downward into the palm of the lid, such that the Rpn11 catalytic site is obscured by Rpn5. In the mature proteasome, the Rpn8–Rpn11 heterodimer undergoes an ~90^o^ rotation upward and out from the palm of the lid, exposing the Rpn11 catalytic site and poising it for deubiquitination of substrates.

Cryo-EM demonstrated that Rpn11 exists in an inhibited form in the isolated lid that explains these biochemical observations (Figure 6) [83]. In the free lid, the N-terminal domain of Rpn5 interacts closely with loops surrounding Rpn11’s catalytic Zn^2+^ ion and also occludes the substrate-binding cleft. This dual mechanism of inhibition is also mediated in part via interactions between Rpn8 and Rpn9 that hold the Rpn8–Rpn11 heterodimer downward against the palm of the hand-like lid subcomplex. Incorporation of the lid into the 26S proteasome results in significant conformational changes, particularly in the position of the Rpn11/Rpn8 domain heterodimer. Upon incorporation into the proteasome, it undergoes an ~90^o^ upward rotation that extends it over the substrate-translocation channel. This transition is likely facilitated by the distortion of Rpn11–Rpn5 and Rpn8–Rpn9 contact sites caused by interactions with the core and base subunits, and/or via displacement of the Rpn8/Rpn11 heterodimer by Rpn10 incorporation. The activation of Rpn11 in the proteasome is further enhanced by the stabilization of an alternative conformation in a key substrate-interacting loop near the active site through interactions with the Rpt4/Rpt5 N-terminal coiled coil domain [69,74,88]. Together, these autoinhibitory interactions among lid subunits serve to restrain the deubiquitinating activity of Rpn11 until it can be safely coupled to the translocation of the substrate through the ATPase pore.

More recently, a related regulatory mechanism for the ATPase activity of the base was described [91]. AAA+ family ATPases, such as the Rpt subunits within the proteasome base, form an ATP-hydrolyzing active site at the interface between the large N-terminal domains of one subunit and the small, C-terminal helical domain in the counterclockwise adjacent subunit [85]. It has been known for some time that the Rpn14/Nas6, Nas2, and Hsm3 modules harbor little to no ATPase activity on their own [26]. However, the fully assembled base displays robust ATPase activity, approximately 50% of that observed for the proteasome holoenzyme [92]. Although it had been assumed that this two-fold stimulation of ATPase activity likely is mediated by interactions between the base and the lid and/or CP, careful measurements of ATPase activity by purified base bound by different combinations of chaperones has demonstrated that they cooperatively suppress the ATPase activity of the base [91].

Although the mechanism by which the assembly chaperones suppress the ATPase activity of the base is unknown, it is tempting to speculate that they may function (at least in part) by constraining the conformational space sampled by the base. Consistent with this possibility, ATP binding and hydrolysis is known to drive large conformational transitions within AAA+ family ATPases; suppression of such transitions in a given subunit would be expected to negatively regulate ATP binding and/or hydrolysis by its neighbors via allosteric effects. Despite this seemingly straightforward model, it is unclear from available structures of Rpn14 [93,94,95], Nas6 [84], or Hsm3 [62,68,96] how they may negatively regulate conformational changes within the base. Nas6 is not anticipated to make any contact with ATPases other than its binding partner Rpt3, and its position on the C-terminal helical domain of Rpt3 does not appear to be suitable for influencing the ATP hydrolysis cycle directly. Hsm3, in contrast, binds Rpt1 such that it also contacts Rpt2, and thus could serve much like a wedge to lock these two subunits into a particular ATP hydrolysis-deficient state [62]. At present, no structure of Rpn14 in complex with its binding partner Rpt6 is available to inform how it may impact its function; structural studies of the base adorned by particular combinations of assembly chaperones will likely be critical for understanding this mechanism of negative ATPase regulation.

### 3.3. Conformational Dynamics as a Means to Evict Assembly Chaperones from Nascent Complexes

Another avenue in which conformational dynamics are exploited during proteasome biogenesis is to ensure the timely dissociation of assembly chaperones upon maturation of their respective client complexes. To ensure efficient proteasome biogenesis, it is important that dedicated assembly chaperones recognize their cognate assembly intermediates specifically and with high affinity, as these highly transient, metastable intermediates are often present at very low steady-state levels in cells. However, this creates a challenge, in that this tightly bound chaperone must dissociate just as efficiently upon maturation of the bound subcomplex. Surprisingly, very few examples of how chaperones are released from nascent proteasomes or subcomplexes exist. However, in known examples, conformational changes play important roles in promoting the release of chaperones.

As described above, the Pba1–2 chaperone complex associates during the initial stages of CP α ring formation and is retained through CP maturation until it is displaced by the RP. A proposed model for this displacement is via conformational changes in the α ring during maturation of the CP peptidase active sites, resulting in a weakened affinity of Pba1–2 for the CP [97]. This model is based in part on observations that Pba1–2 binding to mature CP is highly salt-sensitive, whereas binding to immature CP is highly salt-resistant and thus presumably of higher affinity than that for mature CP [97]. In further support of this model, a series of structural investigations demonstrated that the α rings in 15S precursors and the PHP are much broader than in mature CP [44,45,98]. This broader CP surface allows Pba1–2 to make increased contact with the α ring and presumably increases its affinity compared to mature CP where contact is more limited. Indeed, a structure of Pba1–2 bound to the PHP compared to a structure of Pba1–2 bound to mature proteasomes reveals the α ring contracts during transition from PHP to mature CP. Further, comparison of the Pba1–2 bound to 15S and the Pba1–2 bound to CP revealed that the chaperone adopts a distended orientation with the mature CP that limits its CP contact compared to the 15S [44]. Thus, this conformation-controlled affinity switch enables Pba1-2 to both guide the correct assembly of the α ring and prevent premature docking of the RP onto the CP, but to be released efficiently upon maturation of the proteolytic active sites [97]. 

Although appealing, it is not yet clear whether differences in the breadth of the CP are the cause of Pba1–2 release or a consequence of them. A model for Pba1–2 release that challenges the one raised above can be envisioned based on the structures of several intermediates of the CP revealed in a recent cryo-EM study [44]. In these structures, the N-terminus of Pba1 can be observed making direct contact with the β5 subunit propeptide, as well as the assembly chaperone Ump1. Thus, one alternative model for Pba1–2 eviction would be one in which the cleavage of β subunit propeptides and the subsequent destruction of Ump1 removes critical avid interactions that would normally stabilize the interaction between Pba1–2 and the CP, triggering their dissociation. Further studies will be necessary to distinguish between these two (and other) possibilities.

An important remaining question is whether the RP or some other element actively displaces Pba1–2 from mature CP, or whether RP adventitiously binds during the transient dissociation of Pba1–2, rendering Pba1–2 unable to rebind. The dissociation constant of Pba1–2 for mature CP was estimated at ~1–3 µM [97,98]. Assuming that their association rate constant is in the range of 10^6^–10^8^ M^−1^ sec^−1^ as is common for many protein–protein interactions, Pba1–2 would dissociate from mature CP with a half-life of ~1–100 s, which would be suitably fast for the latter possibility. However, detailed kinetic analyses will be necessary to distinguish between these possible mechanisms of eviction.

**Figure 7:**
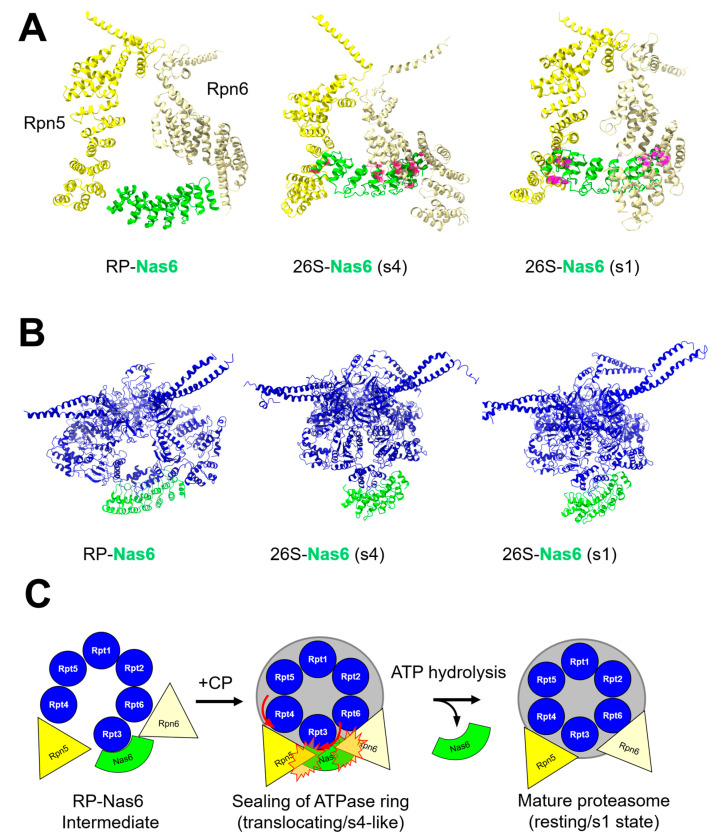
Conformational eviction of Nas6 assembly chaperone from mature proteasomes. (**A**,**B**) the position of the human Nas6 ortholog, gankyrin, bound to the free regulatory particle is shown on the left (PDB: 5VHF). Molecular modeling of Nas6 (PDB: 2DZN) onto the substrate-translocating (s4; PDB: 6FVW; center) or the resting/ground state (PDB: 6FVT) proteasome are shown for comparison. In (**A**), Rpn5 is shown in darker yellow and Rpn6 in lighter yellow, with Nas6 in green. Pink markings indicate positions of steric clash between Nas6 and Rpn5 or Rpn6. Increased steric clash occurs as the proteasomal ATPase ring shifts from the substrate-translocating state to the resting state. In (**B**), the puckering of the ATPase ring is evident when compared to the ATPase ring of the s4 and s1 proteasomes. (**C**) a cartoon model describing the inferred mechanism of Nas6 eviction upon sealing of the ATPase ring and stimulation of hydrolysis at the Rpt3 catalytic pocket.

Much as conformational changes in the maturing CP serve to promote release of Pba1–2, a similar mechanism is utilized to evict one base assembly chaperone, Nas6, from nascent proteasomes. Earlier work by the Park group demonstrated that when bound to the isolated base, Nas6 caused steric clash either with the lid or with the CP, depending on the nucleotide state of the base [86]. Specifically, loading the Nas6-bound base with the non-hydrolyzable ATP analog ATPγS promoted efficient interaction between the base and CP, but inhibited interaction between the base and lid. Conversely, provision of ATP (which is presumably rapidly hydrolyzed to ADP and phosphate by the base) supported efficient interaction between the base and the lid, but inhibited interaction between the base and the CP. Molecular modeling of Nas6 onto mature proteasomes in various conformational states provided a rationale for this observation—the C-terminal helical domain of Rpt3 to which Nas6 binds was differentially positioned in structures of the proteasome generated in the presence of ATP vs. ATPγS. This positioned Nas6 to clash either with the CP surface or the lid subunits Rpn6 [86] and Rpn5 [87] (Figure 7A). Thus, the conformational state of the base can deploy Nas6 in different ways to restrict association between a particular subcomplex.

This observation raises an apparent paradox. Nas6 appears to prevent the formation of a ternary lid•base•CP complex, as it will be in steric clash with either the lid or CP at any given time. Some insights into how this issue may be resolved were provided by a series of cryo-EM structures of the human ortholog of Nas6, p28/gankyrin, bound to the RP [99]. In a subset of structures, the ATPase ring of the base appeared to have adopted a flatter, more open position by virtue of a puckering of the ATPase ring centered on Rpt3 (Figure 7B, left). This puckering would in principle permit the formation of such a ternary complex, with the expectation that closing of the puckered ring would then serve to evict Nas6. Although the importance of this puckering was not validated experimentally, a recent biochemical study has implicated conformational changes in the nascent 26S proteasome as the signal for eviction of Nas6 from the nascent mature proteasome [87]. The use of ATPase-specific mutations and conformation-specific disruptions revealed an ATP-dependent remodeling of Rpn5 that generates enhanced steric clash to evict Nas6 from nascent proteasomes. Specifically, the contact between Rpn5 and the base promoted the eviction of Nas6 in a manner dependent upon the nascent proteasome adopting a resting/s1-like state (Figure 7A,B right panels). Taken together, these three studies yield a model in which the Nas6-bound RP, by virtue of the puckered ATPase ring, forms a ternary Nas6•RP•CP complex. We speculate that, upon formation of this complex, docking the RP to the CP promotes closure of the ATPase ring, bringing Rpn5 into steric conflict with Nas6, leading to its release (Figure 7C).

Consistent with this model, the Park group recently demonstrated that Rpn14, Nas6, and Hsm3 work together coordinately to suppress ATPase activity of the base during assembly [91]. Suppression of ATPase activity would promote the accumulation of unhydrolyzed ATP in the binding sites of the proteasomal ATPases, yielding a conformation of the ATPase ring most consistent with the translocating/s4 state of the proteasome. This conformation was shown to have rather limited steric conflict with Nas6 in modeling studies, whereas the conflict was increased substantially when Nas6 was modeled onto the resting/s1 state of the proteasome [87]. Thus, stimulation of ATP hydrolysis upon docking of the chaperone-bound RP onto the CP would serve to effectively shoehorn Nas6 from the nascent proteasome via the enhanced steric conflict generated as the proteasome shifts from the translocating/s4 state to the resting/s1 state (Figure 7C).

Although this model clearly explains the release of Nas6, it remains largely unclear how the two other RP-associated chaperones, Rpn14 and Hsm3, are released from nascent proteasomes. It is likely that ATP binding and/or hydrolysis are also exploited in the case of these chaperones to drive their eviction from this ternary complex; dissecting the mechanisms as well as the relative sequence of chaperone eviction is likely to yield important insights into the earliest stages of ATPase function within nascent proteasomes and will clarify how these complicated mechanistic steps are accomplished.

### 3.4. Conformational Dynamics to Signal Dysfunctional Proteasomes

In a similar vein to the role of assembly chaperones in suppressing ATPase activity of the free base subcomplex, Nas6 was recently shown to be unique among the chaperones in that it can destabilize proteasomes with defects at the RP–CP interface [100]. Typically, the RP docks onto the CP on one or both ends in part through highly conserved, flexible C-termini tail regions of specific ATPase subunits [67]. These C-termini regions terminate in a 3-amino acid HbYX motifs (where Hb, Y, and X indicate a hydrophobic amino acid, a tyrosine, and any amino acid, respectively) insert themselves into pockets formed at the interfaces between adjacent subunits located on the surface of the α ring of the CP. The tails of Rpt2, Rpt3, and Rpt5 have been visualized to be stably docked in virtually all structures of the proteasome, whereas the tails of Rpt1 and Rpt6 appear more dynamic, being engaged with their cognate α ring pockets primarily in proteasomes that are actively degrading substrates or treated with ATPγS [69,74,76,101].

Disruptions to the HbYX-α pocket interactions on either side of the RP–CP interface yielded a Nas6-dependent reduction in the levels of mature proteasomes and a corresponding increase in the levels of free RP and CP, consistent with a destabilization of this interface [100]. Importantly, this destabilization could be completely rescued via deletion of *NAS6*, indicating it was not simply due to a substantial weakening of the RP–CP interface. Nas6 accumulated selectively on the RP in cells harboring these disruptions, suggesting that Nas6 may either preferentially associate with, or promote the disassembly of, proteasomes with defective RP–CP interfaces. Importantly, this function of Nas6 could be suppressed by artificially tightening the RP–CP interface via addition of ATPγS, suggesting that Nas6 may probe the conformation or dynamics of the RP–CP interface as part of a quality control function taking place after assembly of the proteasome has completed. This surveillance function of Nas6 was proposed to help ensure the quality of the cellular proteasome pool to maintain proteasome homeostasis.

The mechanism by which Nas6 identifies and destabilizes proteasomes with defective RP–CP interfaces remains unclear. It is likely that any defects at the RP–CP interface will weaken the interaction between these complexes; this in principle will enhance the frequency that they dissociate, providing Nas6 opportunities to bind and sequester defective RPs. However, Nas6 accumulated on RPs even when RP–CP interface defects were present on the CP rather than the RP, and deletion of *NAS6* in cells harboring RP–CP defects suppressed the accumulation of free RP and CP, suggesting this former possibility is unlikely [100]. Instead, disruptions to the RP–CP interface may open enough space between these two subcomplexes for Nas6 to bind and sterically interfere with their stable interaction in one or more conformations associated with the ATPase cycle. The molecular signal(s) that Nas6 recognizes, as well as the impacts of this putative quality control function on cell health, will be important to establish in future studies.

## 4. Summary and Perspective

High-resolution structures of the proteasome, combined with biochemical characterization of presumed assembly intermediates, have been instrumental in guiding our understanding of proteasome biogenesis over the past decade. Despite this, virtually all knowledge of proteasome assembly to date has come from static snapshots of the assembly process derived from these biochemical and structural experiments. To fully appreciate how conformational dynamics are harnessed to ensure rapid and faithful assembly of proteasomes, it will be necessary to develop time-resolved and quantitative measurements of the association and dissociation kinetics of key intermediates, as well as the affinities of particular intermediates or subcomplexes for one another. Importantly, these thermodynamic and kinetic measurements must be complemented with real-time reporters of the conformational state of key assembly intermediates to permit a holistic understanding of how structural changes are signaled, and how they regulate key assembly steps. The recent development of approaches to reconstitute proteasomal subcomplexes and intermediates from recombinant proteins [50,92], coupled with site-specific protein labeling technologies [81], will undoubtedly permit the development of such more finely resolved assays and will ensure that the proteasome continues to serve as a foundational model for understanding the assembly of large macromolecular complexes in vivo.

## Data Availability

No new data were created or analyzed in this study. Data sharing is not applicable to this article.
